# Supplementary material for: Functional evolution of SARS-CoV-2 spike protein: Maintaining wide host spectrum and enhancing infectivity via surface charge of spike protein
Source: Comput Struct Biotechnol J. 2023 Mar 12;21:2068–74. doi: 10.1016/j.csbj.2023.03.010 (PMC10008190; doi:10.1016/j.csbj.2023.03.010)

Supplementary

**Figure S1**. Ka/Ks and charge analysis of omicron sublineages(BA.1, BA.2, BA.4, BA.5, BQ.1, XBB) Spike proteins evolution.(A,B) The data was averaged in a day-wise manner. (C) Electrostatic surfaces of the mammalia ACE2 RBD binding site, mouse(7xoc), cat(7c8d) and dog(7e3j) .


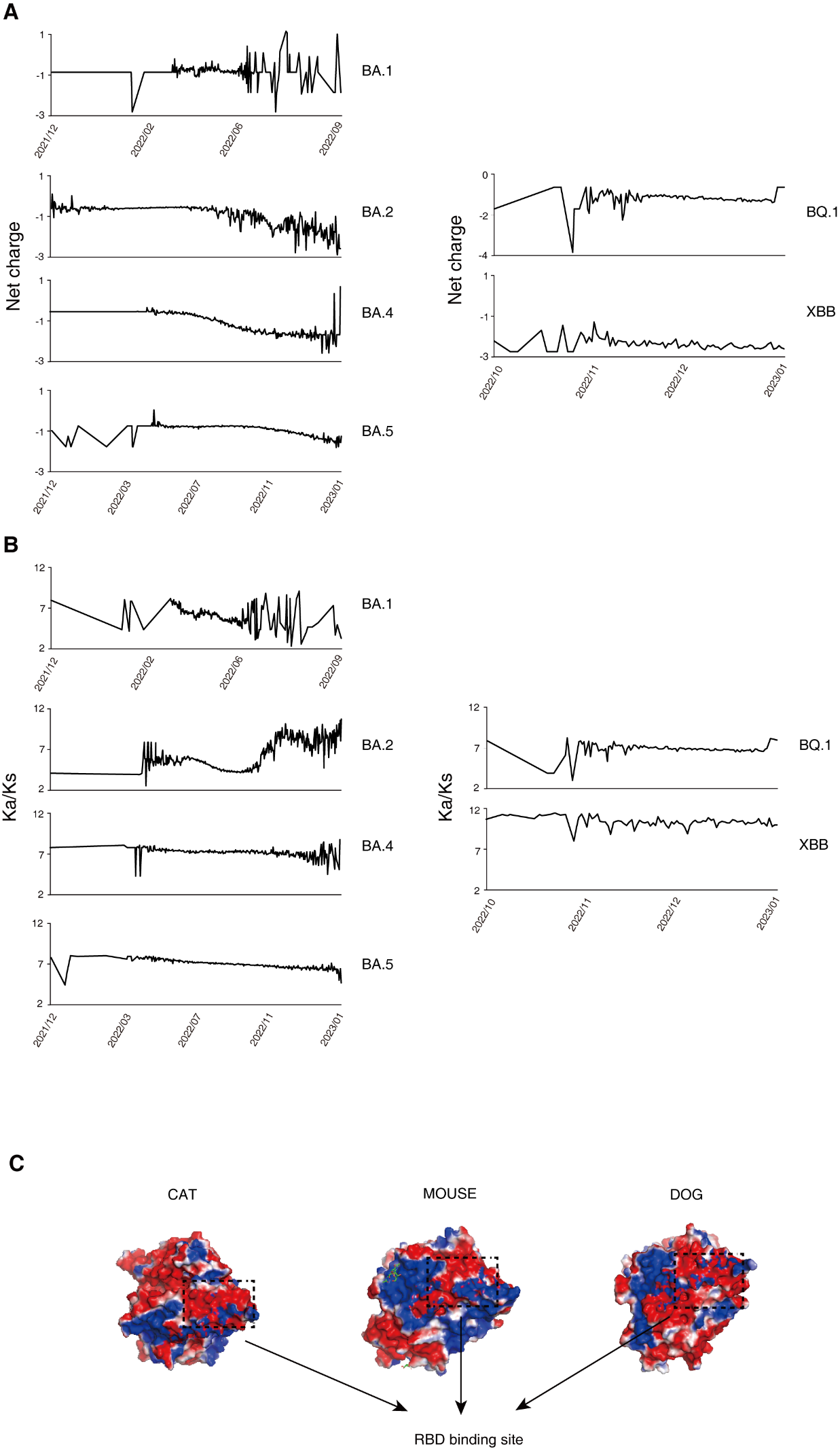

Supplement: Supplementary file 1 — Supplementary material [file mmc1.docx]
